# Estimation of Driver’s Danger Level when Accessing the Center Console for Safe Driving

**DOI:** 10.3390/s18103392

**Published:** 2018-10-10

**Authors:** Hyun-Soon Lee, Sunyoung Oh, Daeseong Jo, Bo-Yeong Kang

**Affiliations:** School of Mechanical Engineering, Kyungpook National University, Daegu 41566, Korea; hslee.knu@gmail.com (H.-S.L.); sunyoung.oh@knu.ac.kr (S.O.); djo@knu.ac.kr (D.J.)

**Keywords:** driver’s danger level, infrared sensor, linear regression analysis, advanced drivers assistance system (ADAS)

## Abstract

This paper proposes a system for estimating the level of danger when a driver accesses the center console of a vehicle while driving. The proposed system uses a driver monitoring platform to measure the distance between the driver’s hand and the center console during driving, as well as the time taken for the driver to access the center console. Three infrared sensors on the center console are used to detect the movement of the driver’s hand. These sensors are installed in three locations: the air conditioner or heater (temperature control) button, wind direction control button, and wind intensity control button. A driver’s danger level is estimated to be based on a linear regression analysis of the distance and time of movement between the driver’s hand and the center console, as measured in the proposed scenarios. In the experimental results of the proposed scenarios, the root mean square error of driver H using distance and time of movement between the driver’s hand and the center console is 0.0043, which indicates the best estimation of a driver’s danger level.

## 1. Introduction

The most important aspect of safe driving is monitoring the driver to prevent the occurrence of serious accidents [1,2,3,4,5]. An advanced driver assistance system (ADAS) [1,2,3,4] is a system for achieving safe driving; the aim of such systems is to reduce the risk of a driver’s accident and assist safe driving. In particular, such a system provides safety to the driver by obviating danger factors while driving [1]. Furthermore, an ADAS supports the automation of control tasks, to relieve the driver from manual control of a vehicle and assist safe driving [5].

Recent research on autonomous vehicles [6,7] has focused on a high-level intelligent ADAS. An intelligent ADAS alerts the driver when the driver’s driving ability is insufficient due to inattention. Safe interface technology between a human and machine is also required to implement an intelligent ADAS. To develop this technology, driver monitoring system (DMS), such as driver status monitoring (DSM) [8,9,10] and driver fatigue monitoring (DFM), are employed to warn the driver about certain dangers [11].

An analysis of the driver’s perception reaction time (PRT) [12,13,14] is required to detect the driver’s state using a DMS. The PRT is an important factor, which directly affects the safety of the driver when driving on the road, and should also be considered in the design of a highway. To monitor a driver’s state, some research [14] has proposed simulator studies, controlled road studies, or naturalistic observation. The advantage of conducting studies using simulators is that it is easy to control such systems for test in experimental environments [8,9,10]. Controlled road studies and naturalistic observation require real-time driving conditions for a driver’s stress measurement [8], while some drivers cannot perform predictive responses for such an experiment. In other words, if an obstacle appears while driving, the result of an experiment needs to show that the driver uses the brake. The driver sometimes avoids an obstacle by driving without using the brake in controlled road studies and naturalistic observation. The reason behind this is that the driver cannot perform predictive response in these cases.

Infrared [15,16,17,18] and ultrasonic sensors [19] are generally used to track driver movements inside a vehicle. It can be difficult to track driver movements using ultrasonic sensors. On the other hand, infrared sensors can be adopted for accurate measurements; these not only keep track of driver movements but they are also economical [15]. Infrared sensors used inside a vehicle detect a driver’s drowsiness [15], and infrared sensors installed outside the vehicle track the movements of other vehicles [16,17].

The driver’s inattention in real-time driving can lead to dangerous accidents. In order to prevent such dangerous accidents and to monitor the experiments on dangerous conditions such as driver’s inattention, the proposed system employs a driver monitoring platform, in view of the advantage of simulator driving. Previous research [15] using an infrared sensor inside the vehicle is limited to detecting a driver’s drowsiness in the driver’s seat. In previous safe driving systems [10,20], the driver’s condition is classified in a discrete manner such as safety and danger. On the other hand, this system proposes a method of estimating the driver’s danger level in a continuous manner from the distance and the time of movement between the driver’s hand and the infrared sensor installed in the center console of the driver monitoring platform. The ground truth value is set for the driver’s danger level in consideration of the distance, time of movement between the driver’s hand and the infrared sensor. Then, the difference was analyzed between the ground truth value of the driver’s danger level and the estimated driver’s danger level using the linear regression analysis [18,21]. Experiments on the driver’s frame dataset collected by eight drivers demonstrate the feasibility of the proposed estimation method.

## 2. Related Work

It is important to monitor the vehicle’s internal and external conditions while driving on the road in order to enhance a driver’s safety [15,16,17]. To detect the motion of an object, a considerable amount of research [15,16,17,18] has employed an infrared sensor, which is an electronic instrument that senses the movement of an object by emitting or detecting infrared rays. Lee et al. [15] proposed a drowsiness detection system based on real-time data of a driver’s head movements using four infrared sensors on the headrest of the driver’s seat and two webcams to record the driver’s state. The driving condition can be classified as normal or drowsy driving based on the driver’s head movement data. In the proposed system, the success rate of detecting drowsy driving was 61% without a learning module. However, the success rate proved to be 78% with a learning module incorporated.

An infrared sensor has been employed on the vehicle body to track the movement or obstacles presented by other vehicles [16,17]. Mobus et al. [16] proposed a driver assistance system called adaptive cruise control (ACC) using an infrared sensor and a 77 GHz radar sensor on the carbody to track other vehicles. Comparing the result of the single sensor tracking algorithm with the result of a multiple sensor fusion algorithm, the authors proved that the multi-sensor fusion algorithm using infrared sensor data and radar data achieved a better detection range and accuracy for object tracking.

Stuckman et al. [17] presented a method for detecting vehicles or objects in a driver’s blind spot using an active infrared sensor. The signal received from the infrared sensor was generalized through filtering, amplification, and rectification. A binary correlation coefficient was applied between the generalized data and received data to detect whether the correlation coefficient was greater than 0.5. Therefore, one of the factors that affected the proposed system was color, and bright objects were easier to detect than dark ones.

Malheiros et al. [18] presented a linear regression study between the beam diameter and the distance of an infrared sensor on an industrial robotic arm. This system employed the infrared distance sensor of Sharp’s GP2Y0A21YK0F model in a Motoman HP6 industrial robot arm with an NX100 controller. A robotic arm and an obstacle were placed facing one another, and 128 output samples were collected while moving at right angles, with the distance decreasing by 5 mm intervals starting from a maximum distance of 80 cm to a minimum distance of 8 cm. The experimental result showed that the measured beam diameter and distance are in the linear relationship with the linear regression slope of 0.0399.

Naqvi et al. [20] developed a gaze detection system for safe driving that classified 17 gaze zones using a convolutional neural network. In order to verify the validity of the method, they designed an experiment including the images of left eye, right eye and face, respectively. The images were collected for 20 drivers including 3 wearing glasses using a near-infrared (NIR) camera considering the driver’s head and eye movement. The accuracy of gaze detection was strictly correct estimation rate (SCER) of 92.8% and loosely correct estimation rate (LCER) of 99.6%, which show good accuracy.

Fernandes et al. [22] predicted risky driving behaviors from risky driving factors using a multiple linear regression model. The research compared a driver’s risky driving behaviors with their risk factors while driving. The first stage examined the risk factors for 109 young drivers. In order to generalize the driver’s risk behaviors of first stage, the second stage selected 115 drivers of various ages, sex, and ethnicity. The second stage tested three analysis methods, and employed full regressions including age as a predictor, regressions excluding age as a predictor, and regressions in an age-restricted sample. As a result of the experiment, it was found that if the age factor was controlled of second stage, two risk factors such as speeding and not wearing seat belts are not generalized as the result of two driver’s risk factors of first stage.

Previous studies using infrared sensors have been limited to detecting drowsy driving using an infrared sensor inside a vehicle or recognizing obstacles using an infrared sensor on the outside of a vehicle. Research on estimation systems using linear regression analysis in a vehicle have also been limited to estimating driver risk behaviors. Therefore, this study employed infrared sensors installed in the center console of a vehicle to detect driver’s movements inside the vehicle. Based on a linear regression analysis, we estimate a driver’s danger level by considering the distance and time of movement between the driver and the center console.

## 3. Frame Dataset and Proposed Methods

**Figure 1:**
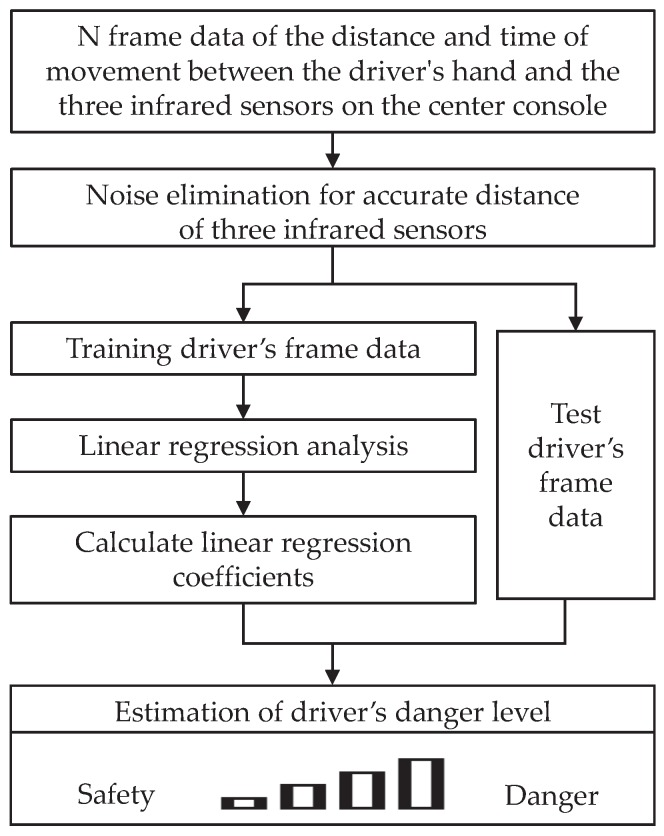
The proposed driver’s danger level estimation approach.

In this paper, a system is proposed for estimating a driver’s danger level by measuring the distance and time of movement between the driver’s hand and the center console during driving. Figure 1 presents an overview of the proposed estimation system. First, three infrared sensors were installed in the center console of the driver monitoring platform. A driver drives the vehicle, and the distance and time of movement between their hand and the three infrared sensors in the straight sections of the proposed driving scenario are measured. In order to collect accurate distance data between the driver’s hand and the center console, the infrared sensor was set to detect the area around the center console, and the noise of the infrared sensor value was filtered. Subsequently, a linear regression analysis trained the collected frame data, consisting of measured distances with time of movement and the ground truth values of a driver’s danger level. When a new test distance and time of movement is given, the same process is performed, and the driver’s danger level is estimated by applying the returned linear regression coefficient.

### 3.1. Accurate Distance Measurement Process

**Figure 2:**
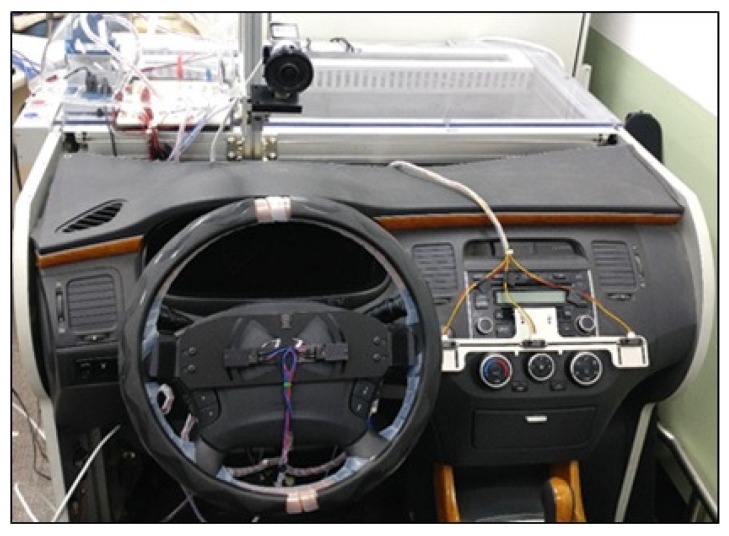
Experimental design on driver monitoring platform.

A driver monitoring platform for the laboratory environment was employed to simulate the exact experimental environment of the driver. This was used to measure the driver’s danger level of the distance and time of movement between the driver and the center console during driving. The driver monitoring platform adopted the Hyundai Grandeur car model. In Figure 2, the infrared sensors installed to collect frame data on the driver’s hand position when accessing the center console of the driver monitoring platform are illustrated. To simulate an actual driving situation in the laboratory, a driver monitoring platform was employed. In this driver monitoring platform, three infrared sensors on the center console were employed to collect the driver’s hand position when accessing the center console.

To measure accurate data for the driver’s hand position, the frame data of the infrared sensors was collected using the Data Acquisition (DAQ) equipment of National Instruments. Then, to monitor the measurement of the accurate distance between the driver’s hand and the infrared sensors collected from the DAQ equipment, a system was developed to collect measured distances from each of the three infrared sensors using the Labveiw program. The system was designed to represent the distance between the driver’s hand and each of the three infrared sensors and the total driving time. As a result, we were able to check the accurate distance and time of movement between the driver’s hand and the three infrared sensors of the center console of the driver monitoring platform using this system in real time.

The module of the infrared sensor for the experiment was the GP2Y0A21YK model by Sharp. The operation principles of a sensor are as follows. The infrared transmitter emits an infrared light to an object’s surface, and the reflected infrared light is then absorbed by the infrared receiver. Then, the voltage value is output by measuring the amount of absorbed infrared light. Finally, the distance between the object and the infrared sensor is measured. In this study, the infrared sensors installed in the center console emitted infrared light to the driver’s hand, and then this infrared light was reflected and absorbed by the infrared module to determine whether the driver’s hand was detected. When the driver’s hand was detected by the infrared sensor, the voltage value was measured.

While collecting driver’s frame data, the infrared sensor’s noise was generated. The raw data for the measured voltage values was difficult to analyze owing to the infrared sensor’s noise. Therefore, the infrared sensor’s noise was filtered to collect precise driver’s frame data. The Butterworth filter, which has a maximally flat magnitude response, was employed to reduce the infrared sensor’s noise and measure an accurate distance. Equation (1) [23] defines the Butterworth filter:

(1) $$N\left(\omega \right)=\frac{1}{\sqrt{1+\omega^{2n}}}$$

Here, N(ω) denotes the Butterworth filter, ω indicates the number of vibrations per second, and *n* is the number of poles. The voltage value at which the noise was filtered by applying Equation (1) was used to calculate the distance between the driver’s hand and the infrared sensor using Equation (2):

(2) $$D=27.86{\left(V\right)}^{-1.15}$$

Here, *D* indicates the distance between the driver’s hand and the infrared sensor, and *V* defines the voltage measurement.

**Figure 3:**
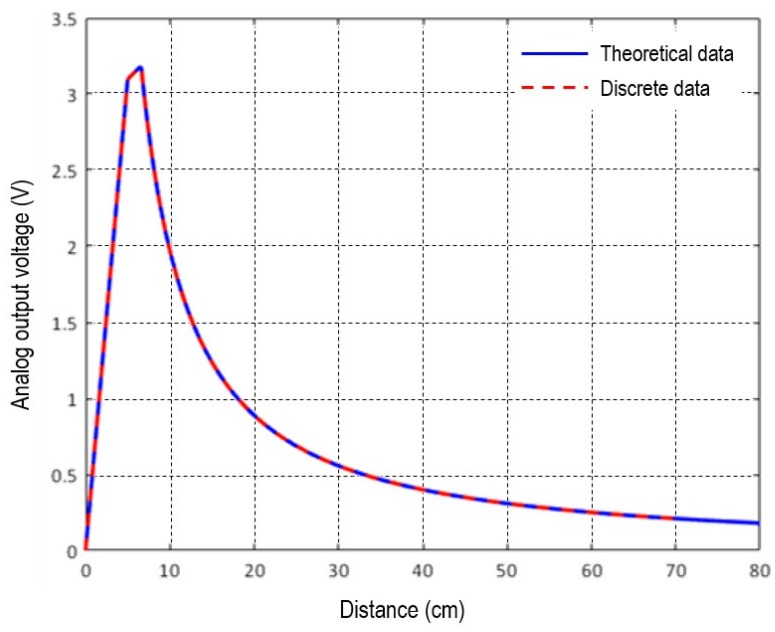
Datasheet for sharp infrared sensor (analog output voltage vs. distance to reflective object).

The infrared module GP2Y0A21YK has a data sheet [24] that is shown in Figure 3. In this datasheet, the infrared module voltage is a minimum of 0.4 V and a maximum of 3.25 V, and its measureable distance is a minimum of 8 cm and maximum of 80 cm as theoretical data. However, after applying the Butterworth filter the results shown by dots in Figure 3 were obtained. The voltage value was exactly the same as for the theoretical data sheet, but the distance was different, in that the minimum value was reduced from 8 cm to 4 cm, and the maximum value was decreased from 80 cm to 69 cm. Therefore, the distance measurement range for the infrared sensors of the driver monitoring platform can be measured from a minimum distance of 4 cm to a maximum distance of 69 cm.

### 3.2. Method of Collecting Driver’s Frame Data

**Figure 4:**
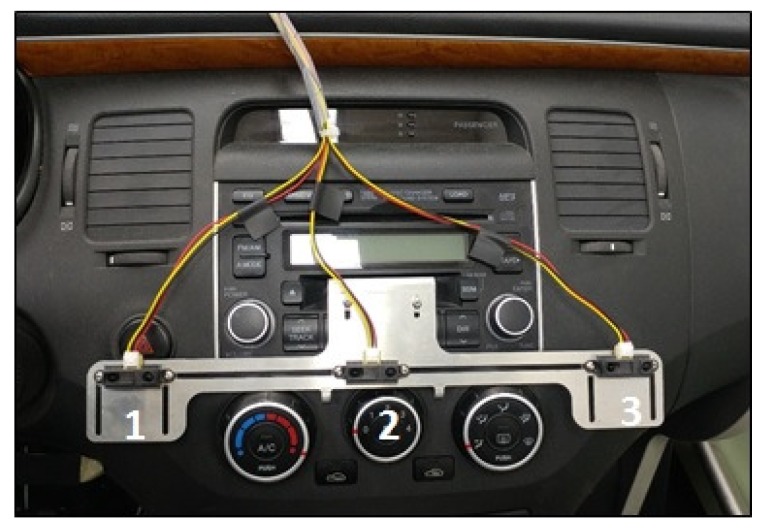
Positions of infrared sensors on the center console.

The three infrared sensors were employed on the center console of the driver monitoring platform, as shown in Figure 4. These three infrared sensors of the center console used in the experiment were installed: at the top of the buttons that control the air conditioner and heater (temperature controller), wind direction controller, and wind intensity controller. The three infrared sensors were installed on the center console in an x-axial direction from the center of the steering wheel at distances of 22 cm, 36 cm, and 54 cm. The position of the right end of the center console between the driver and passenger is 22 cm from the steering wheel, which is defined as 〈1〉. The middle position, representing the wind direction control button, is 36 cm, and is set as 〈2〉. The position on the left end is 54 cm, and is defined as 〈3〉.

**Figure 5:**
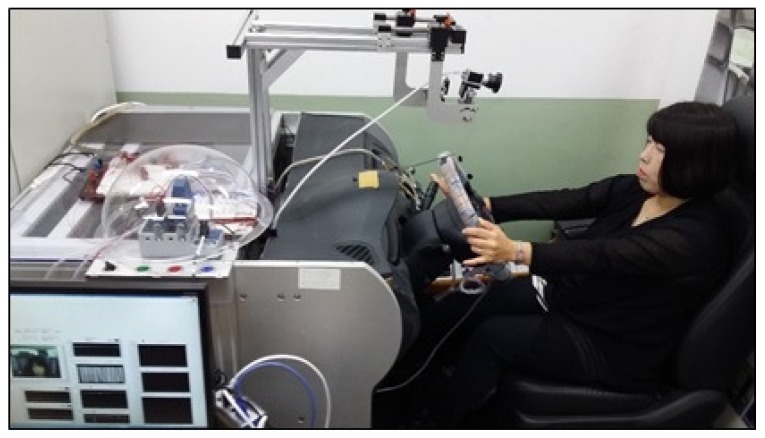
Experimental scenario for collecting access frame data for the center console of the driver monitoring platform.

The experimental environment for collecting the driver’s frame data on the distance between the driver’s hand and the center console is shown in Figure 5. As shown in the figure, one participant sat in the driver’s seat of the driver monitoring platform and drove using the proposed scenarios. Using the system implemented in the Labview 2014 program, the driver’s frame data was collected. A total of eight drivers participated in the experiments under the proposed scenarios, and the driver’s frame data were collected on the distances and time of movement between each driver’s hand and the infrared sensors while driving straight ahead. The participants of the eight drivers, who have a Korean driver’s license, had driving experiences from a minimum of three months to a maximum of 20 years. Of these participants, two drivers were Chinese and six drivers were Korean, four drivers were female and four drivers were male. The average age of the eight voluntary participant drivers was 31 years.

**Figure 6:**
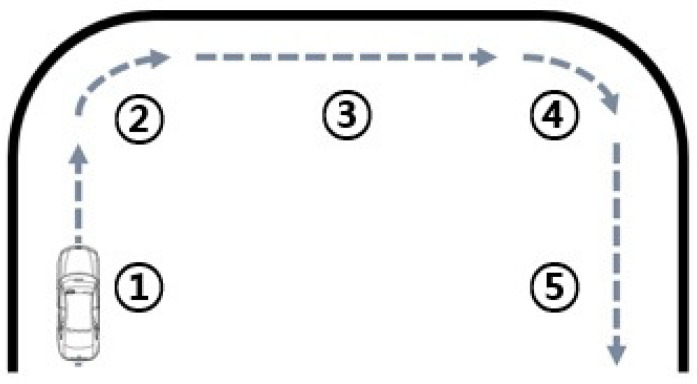
Proposed virtual road condition (turning right).

In order to collect the driver’s frame data for the distance and time of movement between the driver’s hand and the infrared sensors, a virtual road was defined. The virtual road included five driving sections such as three go straight sections and two turn right sections, as depicted in Figure 6. The total driving time for each of the scenarios was 60 s on the virtual road, as detailed in Table 1. The numbered lists of the proposed driving conditions are as follows:

1. Go straight for 17 s.
2. Turn right for 3 s.
3. Go straight for 20 s.
4. Turn right for 3 s.
5. Go straight for 17 s.

Table 2 shows the eight proposed scenarios for each of the eight drivers. In addition, Table 2 details the proposed scenarios for estimating the driver’s danger level using the distance and time of movement between the driver’s hand and the infrared sensors while driving on the straight sections (①, ③, ⑤ in Table 1). To set the distance, the distances between the driver’s hand and infrared sensors while driving straight ahead are set to be close to the minimum measurable distance. In the right turning sections (②, ④ in Table 1), the maximum distance is measured, because the driver’s hand does not move towards the infrared sensors. Therefore, the time corresponding to the driver’s danger level is not measured. To employ the time with the distance, the time is set as either short or long, because the driver’s danger level differs depending on whether their hand accesses the center console for a long or short time while driving. Therefore, the proposed scenarios consist of the cases of “being close for a short time” and “being close for a long time.” To classify between the short and long time, the reference value is set to be 2.5 s, which represents the perception reaction time (PRT) [12,13,14]. The reference value means total time measured by one movement in one section.

To measure the various patterns of driver’s danger level, the proposed scenarios considered repetitive motions in which the driver’s hand moved closer to the infrared sensor 〈1〉 of the three infrared sensors and remained there for either less than 2.5 seconds or longer than 2.5 seconds. The proposed scenarios were performed to each of the infrared sensors 〈2〉 and 〈3〉 in turn. In scenario III of Table 2, driver A’s hand repeats the motion of moving close to the infrared sensor 〈1〉 of the three infrared sensors for less than 2.5 s four times during the 17 s driving time on the first straight section (①). Furthermore, during the 20 s driving time of the second straight section (③), driver A’s hand repeats the motion of moving closer to the infrared sensor 〈1〉 for over 2.5 s three times. During the 17 s driving time for the third straight section (⑤), driver A’s hand repeats the motion of moving close to the infrared sensor 〈1〉 for less than 2.5 s four times. Then, the distance and time of movement between driver A’s hand and infrared sensors 〈2〉 was measured using the same process. The remaining infrared sensors 〈3〉 were also measured. Therefore, we collected frame data consisting of the distance and time of movement between driver A’s hand and infrared sensors 〈1〉, 〈2〉 and 〈3〉 using the process of this scenario III.

Each scenario, considering the distances and time of movement between the driver’s hand and the three infrared sensors of the center console in the straight sections (①, ③, ⑤) represents data on a total of 300 frames collected at a rate of 5 frames per second (fps). Table 3 shows the sample frame used in the experiment that driver A collected in the straight section (⑤) according to scenario III for infrared sensor 〈1〉. Moreover, a total of 85 frame data including omitted data is shown. Here, the omitted data and the same distance data from the three infrared sensors (216, 233, 241, 254, 261, 273, 280, and 294) means that driver A’s hand does not move towards any of the three infrared sensors. Thus, the values of the three infrared sensors are the maximum distances, and the time of movement is zero s. As shown in Table 3, the sample frame data of driver A is the case in which the driver A’s hand approaches the infrared sensor 〈1〉 four times. One frame consists of the distance between driver A’s hand to infrared sensor 〈2〉, and infrared sensor 〈3〉 being the same at 69 cm. It also consists of the time of movement between driver A’s hand at 1.2 s and the distance from hand to infrared sensor 〈1〉 being at 6.76232 cm. For example, between the frames 295 to 300, driver A’s hand moves once towards the infrared sensor 〈1〉 for less than 2.5 s. That is, the total time of the movement of driver A’s hand towards the infrared sensor 〈1〉 is 1.2 s. The value of the infrared sensor 〈1〉 represents the distance of the driver’s hand to the infrared sensor 〈1〉 for 1.2 s. The infrared sensors 〈2〉 and 〈3〉 are 69 cm, which means the maximum distance, respectively. The total number of frames used for the experiment consists of the frames merged sequentially for the data from scenarios I through VIII, giving data on a total of 7200 frames.

### 3.3. Approach to Estimating the Driver’s Danger Level

After the driver’s frame data were collected for the proposed driving scenarios, a linear regression analysis [18,21] was applied to estimate the driver’s danger level for a close distance and time between the driver’s hand and the infrared sensors 〈1〉, 〈2〉, and 〈3〉 installed on the center console.

Linear regression analyses [21] can be classified into simple linear regression analyses, in which there is one predictor variable, and multiple linear regression analyses, in which there are several predictor variables. In this paper, a multiple linear regression analysis is employed, because several predictor variables are considered. To estimate the driver’s danger level, the linear regression analysis Equation (3) is applied.

(3) $$ev_{i}=\beta_{0}+\beta_{1}u_{i1}^{IR1}+\beta_{2}u_{i2}^{IR2}+\beta_{3}u_{i3}^{IR3}+\beta_{4}u_{i4}^{Time},i=1,2,\ldots l$$

Here, $u^{IR1}$, $u^{IR2}$, and $u^{IR3}$ denote the distance between the driver’s hand and infrared sensor 〈1〉, 〈2〉, and 〈3〉, respectively. $u^{Time}$ denotes the time of movement between the driver’s hand and the three infrared sensors. ev represents the estimated value of the driver’s danger level in test driver’s frame data, β denotes the coefficient of the linear regression analysis estimated by the linear regression analysis, and *l* is defined as the number of driver’s frame data.

The linear regression analysis employed in this paper represents how close to linear the relationship is between the four predictor variables $u^{IR1}=\left[u_{1}^{IR1},u_{2}^{IR1},\ldots ,u_{l}^{IR1}\right]$, $u^{IR2}=\left[u_{1}^{IR2},u_{2}^{IR2},\ldots ,u_{l}^{IR2}\right]$, $u^{IR3}=\left[u_{1}^{IR3},u_{2}^{IR3},\ldots ,u_{l}^{IR3}\right]$, and $u^{Time}=\left[u_{1}^{Time},u_{2}^{Time},\ldots ,u_{l}^{time}\right]$, and the response variables $ev=[ev_{1},ev_{2},\ldots ,ev_{l}]$. Therefore, β represents the estimated value of the linear regression coefficient for the multiple linear regression analysis of ev, which is a response variable for the predictor variable *u*.

Thus, in training driver’s frame data, the predicted variables represent the distances and time of movement between the driver’s hand and the three infrared sensors, and the response variables represent the ground truth values of the driver’s danger level. Based on the linear regression coefficient estimation β calculated in this process, the estimated value of the driver’s danger level can be calculated for the frame data consisting of the distance and time of movement between the driver’s hand and the three infrared sensors to be tested.

### 3.4. Experimental Results

The ground truth value for the driver’s danger level were set by considering the distances and time of movement between the driver’s hand and the center console’s three infrared sensors employed in the experiment. The case in which the driver’s hand does not get close to the center console is defined as “safety.” In addition, the ground truth value of “safety” is set to zero. The case in which the driver’s hand is close to the center console is defined as “danger.” First, the ground truth value for the driver’s danger level is defined considering the time of movement between the driver’s hand and the center console. The longer the time of movement, the higher the driver’s danger level. On the other hand, the shorter the time of movement, the lower the driver’s danger level. Therefore, the ground truth value for the time of movement is defined as a value that increases by 0.1 in proportion to the driver’s level if the time increases by 0.2 s (5 fps) intervals. Then, the ground truth value for the driver’s danger level is defined considering the distance between the driver’s hand and the center console. The closer the distance between the driver’s hand and the center console, the higher the driver’s danger level. On the other hand, the farther the driver’s hand is from the center console, the lower the driver’s danger level. The ground truth value for the distance is defined as (1/distance) because the driver’s level is inversely proportional to the distance. Thus, the ground truth value for the driver’s danger level combining the distance and time of movement is defined as a value that adds (1/distance) to the number of data in increasing time × 0.1. For example, in the case that the distance between the driver’s hand and infrared sensor 〈1〉 is 4 cm and the measured time is 0.2 s, the driver’s danger level is 0.1, and when the reciprocal of 4 is added, the driver’s danger level becomes 0.35. For the case in which the driver’s danger level is higher than a certain numerical value, an alarm can be set up.

Table 4 presents the experimental results based on the linear regression analysis for the frame data consisting of the ground truth values for driver’s danger level and the distances and time of movement between the driver’s hand and the three infrared sensors for eight drivers for each scenario in Table 2, i.e., scenarios I through VIII. Each of the proposed scenarios was performed for the following proposed methods: a method considering the distances and times between the driver’s hand and the infrared sensors, one considering the distances only, and one considering the times only. Leave-one-out cross validation (LOOCV) and 10-fold cross validation were employed to validate the performance of the frame data using the linear regression analysis. Suppose that the total number of entire data is *N*. Then, LOOCV is a method of using N−1 training data in order for one data point to be tested. Furthermore, 10-fold cross validation is a method of dividing the total of *N* data into 10 equal parts, then employing each single part one-by-one as the test data with the remaining nine parts as the training data.

With respect to each proposed method and the validation methods, the root mean square error (RMSE) was calculated for the driver’s danger level. The RMSE is used to handle the difference between the estimated value and the ground truth one. If the RMSE is large, then the error is large, and vice versa. The RMSE used in the experiment is defined in Equation (4):

(4) $$r=\sqrt{\frac{\sum_{k=1}^{T}{\left(\omega_{k}-{\hat{\omega }}_{k}\right)}^{2}}{T}}$$

Here, *T* is defined as the total number of frame data, $\omega_{k}$ means the ground truth value of an arbitrary frame data, and ${\hat{\omega }}_{k}$ denotes the value estimated using the linear regression analysis for an arbitrary frame data. For example, the RMSE of scenario VII is 0.4671 when estimated the driver’s danger level by using the LOOCV method and the method considering only the distances between the driver’s hand and the three infrared sensors. On the other hand, in the case of using the LOOCV method and both the distances and times between the driver’s hand and the infrared sensors for the same scenario, the RMSE is 0.0068. Therefore, it is found that the frame data that considers both the distances and times between the driver’s hand and the center console exhibits a lower RMSE than that considering only the distances. Under the same conditions, comparing the result obtained when using both the distances and times with the result obtained when using the times only, the RMSE of estimating the driver’s danger level when using only the times for the driver’s hand being close to the infrared sensors is 0.0520, which is larger than the RMSE using both the distances and times. The other scenarios exhibit the best RMSE results when considering the times and distances between the driver’s hand and the infrared sensors. Therefore, for all the proposed scenarios it is determined that when both the distances and times between the driver’s hand and the infrared sensors are considered together, which reflects the proposed method, the best estimation of the driver’s danger level is obtained.

Table 5 presents the test results of the total frame data in all the scenarios for each of the eight drivers. In the case of considering both the distances and time of movement between driver A’s hand and three infrared sensors for all scenarios and employing the LOOCV method, the RMSE is 0.0049. On the other hand, for the same method, the RMSE is 0.3096 when only the distances are considered, and 0.0466 when only the times are considered. The RMSEs considering the distances and the times for the total frame data for all drivers exhibited the best results. Therefore, the approach considering the distances and times together for the total frame data for all drivers provides the best estimation of the driver’s danger level. Hence, the proposed method provides accurate and detailed estimations of the driver’s danger level.

Comparison of the proposed method with previous research for driver’s safe driving are summarized in Table 6. In [10] a drowsiness detection system of fuzzy Bayesian network considering smartphone, electrocardiography (ECG) shows true awake of 96%, true drowsy of 97%. In [20], a gaze detection system was implemented as convolutional neural network (CNN) using a near-infrared (NIR) camera. This system shows strictly correct estimation rate (SCER) of 92.8% and loosely correct estimation rate (LCER) of 99.2%. The proposed system shows the RMSE of 0.0043 in Table 5, which means the best estimated result of the driver’s danger level. Therefore, the previous systems can be classified as driver’s safety or driver’s danger but the proposed system estimates the driver’s danger level. Moreover, the performance of the proposed method is not directly comparable with the performance of the two previous research studies because the performance comparison method is different.

## 4. Conclusions

In this study, three infrared sensors were installed on the driver monitoring platform to estimate the driver’s danger level by considering a close distance and time of movement between the driver’s hand and the center console. Then the frame data for the distance and time of movement between the driver’s hand and the three infrared sensors were collected under the proposed scenarios. The driver’s danger level was estimated based on the linear regression analysis. As a result, by analyzing the RMSE for the proposed methods, the case in which both the close distance and time of movement between the driver’s hand and the center console were considered yielded the best results. Therefore, it is found that a detailed and accurate estimation of the driver’s danger level is provided in this case. However, drivers did not succeed unless they focused on repeating the same movement in one go straight section for approximately 2.5 s using the proposed scenarios in the experiment. Most drivers felt they were partaking in dangerous driving by repeating the same movement for 17 or 20 s. A device for securing the safety of the driver was required when applying the proposed scenarios to the actual driving of the vehicle.

In the future, a comprehensive study for estimation of driver’s danger level will be necessary to consider not only the driver’s images, but also other types of sensor data collected on the driver monitoring platform. It should also be considered whether the driver’s mobile phone or the center console’s touch screen will be used while driving.

## Figures and Tables

**Table 1:** Proposed driving conditions and the measured distance between a driver’s hand and the three infrared sensors of the center console for each route in the virtual road condition. (s: seconds)

Route No.	①	②	③	④	⑤
Distance	Close	N/A	Close	N/A	Close
Driving direction	Go straight	Turn right	Go straight	Turn right	Go straight
Driving time	17 s	3 s	20 s	3 s	17 s

**Table 2:** Proposed scenarios for measuring the distance and time of movement between a driver’s hand and the three infrared sensors while driving straightfor each of the eight drivers. (s: seconds, The number of hand colse ups: the number of driver A’s hand close ups, 〈1〉: infrared sensor 〈1〉, 〈2〉: infrared sensor 〈2〉, 〈3〉: infrared sensor 〈3〉)

Proposed Scenario	①. Go Straight (17 s) Close Distance	③. Go Straight (20 s) Close Distance	⑤. Go Straight (17 s) Close Distance
I	Repeat more than 2.5 s	Repeat more than 2.5 s	Repeat more than 2.5 s
The number of hand close ups	〈1〉: 3, 〈2〉: 3, 〈3〉: 3	〈1〉: 3, 〈2〉: 3, 〈3〉: 3	〈1〉: 3, 〈2〉: 3, 〈3〉: 3
II	Repeat less than 2.5 s	Repeat less than 2.5 s	Repeat less than 2.5 s
The number of hand close ups	〈1〉: 4, 〈2〉: 4, 〈3〉: 4	〈1〉: 4, 〈2〉: 4, 〈3〉: 4	〈1〉: 4, 〈2〉: 4, 〈3〉: 4
III	Repeat less than 2.5 s	Repeat more than 2.5 s	Repeat less than 2.5 s
The number of hand close ups	〈1〉: 4, 〈2〉: 4, 〈3〉: 4	〈1〉: 3, 〈2〉: 3, 〈3〉: 3	〈1〉: 4, 〈2〉: 4, 〈3〉: 3
IV	Repeat more than 2.5 s	Repeat less than 2.5 s	Repeat more than 2.5 s
The number of hand close ups	〈1〉: 3, 〈2〉: 3, 〈3〉: 3	〈1〉: 4, 〈2〉: 4, 〈3〉: 4	〈1〉: 3, 〈2〉: 3, 〈3〉: 3
V	Repeat less than 2.5 s	Repeat less than 2.5 s	Repeat less than 2.5 s
The number of hand close ups	〈1〉: 4, 〈2〉: 4, 〈3〉: 4	〈1〉: 5, 〈2〉: 4, 〈3〉: 4	〈1〉: 3, 〈2〉: 3, 〈3〉: 3
VI	Repeat more than 2.5 s	Repeat more than 2.5 s	Repeat less than 2.5 s
The number of hand close ups	〈1〉: 3, 〈2〉: 3, 〈3〉: 3	〈1〉: 3, 〈2〉: 3, 〈3〉: 3	〈1〉: 4, 〈2〉: 4, 〈3〉: 5
VII	Repeat more than 2.5 s	Repeat less than 2.5 s	Repeat less than 2.5 s
The number of hand close ups	〈1〉: 3, 〈2〉: 3, 〈3〉: 3	〈1〉: 4, 〈2〉: 4, 〈3〉: 4	〈1〉: 4, 〈2〉: 4, 〈3〉: 4
VIII	Repeat less than 2.5 s	Repeat more than 2.5 s	Repeat more than 2.5 s
The number of hand close ups	〈1〉: 4, 〈2〉: 4, 〈3〉: 4	〈1〉: 3, 〈2〉: 3, 〈3〉: 3	〈1〉: 3, 〈2〉: 3, 〈3〉: 3
Total number of hand close ups	〈1〉: 28, 〈2〉: 28, 〈3〉: 28	〈1〉: 26, 〈2〉: 25, 〈3〉: 25	〈1〉: 25, 〈2〉: 25, 〈3〉: 25

**Table 3:** Sample of the collected driver A’s frame data used in the experiment. (scenario III, the straight section (⑤), Infrared sensor 〈1〉: 4)

Number	Infrared Sensor 〈1〉	Infrared Sensor 〈2〉	Infrared Sensor 〈3〉	Time of Movement
216	69.00000	69.00000	69.00000	0.0
⋮	⋮	⋮	⋮	⋮
233	69.00000	69.00000	69.00000	0.0
234	6.30972	69.00000	69.00000	0.2
235	6.36629	69.00000	69.00000	0.4
236	6.53602	69.00000	69.00000	0.6
237	6.30972	69.00000	69.00000	0.8
238	6.19657	69.00000	69.00000	1.0
239	6.30972	69.00000	69.00000	1.2
240	6.36629	69.00000	69.00000	1.4
241	69.00000	69.00000	69.00000	0.0
⋮	⋮	⋮	⋮	⋮
254	69.00000	69.00000	69.00000	0.0
255	6.30972	69.00000	69.00000	0.2
256	6.25314	69.00000	69.00000	0.4
257	6.30972	69.00000	69.00000	0.6
258	6.30972	69.00000	69.00000	0.8
259	6.19657	69.00000	69.00000	1.0
260	6.36629	69.00000	69.00000	1.2
261	69.00000	69.00000	69.00000	0.0
⋮	⋮	⋮	⋮	⋮
273	69.00000	69.00000	69.00000	0.0
274	6.25314	69.00000	69.00000	0.2
275	6.36629	69.00000	69.00000	0.4
276	6.36629	69.00000	69.00000	0.6
277	6.25314	69.00000	69.00000	0.8
278	6.36629	69.00000	69.00000	1.0
279	6.36629	69.00000	69.00000	1.2
280	69.00000	69.00000	69.00000	0.0
⋮	⋮	⋮	⋮	⋮
294	69.00000	69.00000	69.00000	0.0
295	6.25314	69.00000	69.00000	0.2
296	6.25314	69.00000	69.00000	0.4
297	6.30972	69.00000	69.00000	0.6
298	6.36629	69.00000	69.00000	0.8
299	6.30972	69.00000	69.00000	1.0
300	6.76232	69.00000	69.00000	1.2

**Table 4:** Results for the RMSE based on the linear regression analysis of the eight drivers’ frame data for each proposed scenario. (RMSE: the root mean square error, time: time of movement)

Proposed Method	Cross Validation	Scenario							
		I	II	III	IV	V	VI	VII	VIII
Distance & time	LOOCV	0.0071	0.0072	0.0074	0.0069	0.0076	0.0079	0.0068	0.0088
	10-fold	0.0074	0.0073	0.0079	0.0071	0.0080	0.0083	0.0072	0.0093
Distance only	LOOCV	0.5184	0.1107	0.4554	0.5390	0.4240	0.5759	0.4671	0.5593
	10-fold	0.5513	0.1232	0.5337	0.5395	0.4680	0.6127	0.5140	0.6323
Time only	LOOCV	0.0484	0.0329	0.0505	0.0505	0.0891	0.0517	0.0520	0.0512
	10-fold	0.0447	0.0294	0.0475	0.0468	0.0513	0.0486	0.0542	0.0478

**Table 5:** Results of the RMSE for each of the eight drivers in all the scenarios based on the proposed methods. (RMSE: the root mean square error, time: time of movement)

Proposed Method	Cross Validation	Driver							
		A	B	C	D	E	F	G	H
Distance & time	LOOCV	0.0049	0.0212	0.0083	0.0059	0.0107	0.0072	0.0078	0.0043
	10-fold	0.0050	0.0232	0.0083	0.0060	0.0107	0.0072	0.0080	0.0043
Distance only	LOOCV	0.3096	0.6327	0.3881	0.5400	0.6918	0.7695	0.4653	0.2585
	10-fold	0.3109	0.6339	0.3910	0.5434	0.7025	0.7749	0.4708	0.2594
Time only	LOOCV	0.0466	0.0602	0.0502	0.0499	0.0555	0.0584	0.0544	0.0394
	10-fold	0.0466	0.0606	0.0503	0.0500	0.0557	0.0584	0.0545	0.0395

**Table 6:** Comparison of the proposed method with the previous research for safe driving. (CNN: Convolutional neural network, SCER: strictly correct estimation rate, LCER: loosely correct estimation rate, ECG: electrocardiography, PPG: photoplethysmography)

System	Sensor	Method	Goal	Result
Previous [10]	Smartphone,	Fuzzy bayesian	Drowsiness	True awake: 96%
	ECG, PPG	network	detection	True drowsy: 97%
Previous [20]	Near-infrared	Deep learning	Gaze	SCER: 92.8%
	(NIR) camera	(CNN)	detection	LCER: 99.6%
Proposed	Infrared	Linear regression	Estimation of	RMSE: 0.0043
	sensor	anaysis	driver’s danger level	(the best)
